# Matrix Metalloproteinase-2 and -9 Secreted by Leukemic Cells Increase the Permeability of Blood-Brain Barrier by Disrupting Tight Junction Proteins

**DOI:** 10.1371/journal.pone.0020599

**Published:** 2011-08-17

**Authors:** Saran Feng, Jiannong Cen, Yihong Huang, Hongjie Shen, Li Yao, Yuanyuan Wang, Zixing Chen

**Affiliations:** 1 Leukemia Research Unit, Jiangsu Institute of Hematology, 1st Affiliated Hospital, Soochow University, Key Laboratory of Thrombosis and Hemostasis Ministry of Health, Suzhou, China; 2 Department of Hematology, Affiliated Hospital, Xuzhou Medical College, Xuzhou, China; 3 Department of Hematology, Xuzhou Central Hospital, Xuzhou, China; Beth Israel Deaconess Medical Center, United States of America

## Abstract

Central nervous system (CNS) involvement remains an important cause of morbidity and mortality in acute leukemia, the mechanisms of leukemic cell infiltration into the CNS have not yet been elucidated. The blood-brain barrier (BBB) makes CNS become a refugee to leukemic cells and serves as a resource of cells that seed extraneural sites. How can the leukemic cells disrupt this barrier and invasive the CNS, even if many of the currently available chemotherapies can not cross the BBB? Tight junction in endothelial cells occupies a central role in the function of the BBB. Except the well known role of degrading extracellular matrix in metastasis of cancer cells, here we show matrix metalloproteinase (MMP)-2 and -9, secreted by leukemic cells, mediate the BBB opening by disrupting tight junction proteins in the CNS leukemia. We demonstrated that leukemic cells impaired tight junction proteins ZO-1, claudin-5 and occludin resulting in increased permeability of the BBB. However, these alterations reduced when MMP-2 and -9 activities were inhibited by RNA interference strategy or by MMP inhibitor GM6001 in an *in vitro* BBB model. We also found that the disruption of the BBB in company with the down-regulation of ZO-1, claudin-5 and occludin and the up-regulation of MMP-2 and -9 in mouse brain tissues with leukemic cell infiltration by confocal imaging and the assay of *in situ* gelatin zymography. Besides, GM6001 protected all mice against CNS leukemia. Our findings suggest that the degradation of tight junction proteins ZO-1, claudin-5 and occludin by MMP-2 and -9 secreted by leukemic cells constitutes an important mechanism in the BBB breakdown which contributes to the invasion of leukemic cells to the CNS in acute leukemia.

## Introduction

The central nervous system (CNS) is one of the most frequent extramedullary locations in acute leukemia (AL). The prognosis of patients with isolated or mixed CNS relapse is particularly poor. Despite of all kinds of morden therapies (systemic chemotherapy and cranial irradiation) it has become a major obstacle to curing acute leukemia [1]–[5]. Despite its clinical importance, how leukemic cells enter the CNS is poorly understood. The characteristic structure of the blood-brain barrier (BBB) causes the CNS to serve as a resource of cells that seed extraneural sites. But much needs to be learned about how can the leukemic cells disrupt this barrier and invasive the CNS, even if many of the currently available chemotherapies cannot cross the BBB. Moreover, little is known about cellular response of cerebral endothelial cells during invasion of leukemic cell to the CNS.

It is known that the BBB breakdown is associated with brain tumors and diseases of the central nervous system. Although the disruption of the extracellular matrix (ECM) can lead to increased permeability of the BBB in pathological states [6], it is primarily the tight junction (TJ) between adjacent brain microvascular endothelial cells (BMVECs) that confers low paracellular permeability and high electrical resistance, making the barrier to function 50∼100 times tighter than peripheral microvessels [7]–[9]. TJs are composed of transmembrane molecules (claudin, occludin and junctional adhesion molecules) linked to the actin cytoskeleton through cytoplasmic accessory proteins, including zonula occludens (ZO)-1, ZO-2 and ZO-3 [10], [11]. Claudin-5 and occludin were identified as key components of BBB integrity which are localized at the leading edge of BMVECs. ZO-1 acts as a crucial central regulator of the structural organization of the TJ at the plasma membrane [11]. While the barrier and fence functions of TJ have been well understood, it is only relatively recently that the association between TJ and metastasis of cancer cells has been recognized. It is becoming increasingly clear that the TJs have a vital role in maintaining cell to cell integrity and that the loss of cohesion of the structure can lead to invasion and thus metastasis of cancer cells [12], [13].

Some members of the matrix metalloproteinase (MMP) family like MMP-2 and -9 are known to be associated with tumor growth and metastasis because of their capacity to degrade collagen IV, the major ECM component [14]. Earlier reports show an association of MMP-2/-9 expression with invasive behaviour of leukemic cells in acute lymphoblastic leukemia (ALL) and acute myelogenous leukemia (AML) [14]–[17]. Moreover, MMP-2/-9, appears to have prognostic impact in AML [16]. Considerable evidence has accumulated that in some CNS diseases MMP-2 and -9 are involved in the permeability of the BBB by disrupting the junction complexes such as occludin and claudin-5 [18]–[21]. We have thus hypothesized that MMP-2 and -9 secreted by leukemic cells may play critical roles in the BBB opening in CNS leukemia by disrupting TJ proteins.

In order to investigate this hypothesis, we examined the relationship of MMP-2 and -9 secreted by leukemic cells with the TJ proteins and the BBB dysfunction in an *in vitro* model of BBB and in an animal model of CNS leukemia. We show that leukemic cell-derived MMP-2 and -9 disrupt the key TJ proteins ZO-1, claudin-5 and occludin and that this is correlated with the BBB opening. Down-regulation of MMP-2 and -9 secreted by leukemic cells reduces the disruption of these three proteins with decreased permeability of the BBB and also protect mice against CNS leukemia. These data implicate MMP-2 and -9-mediated down-regulation of TJ proteins as a significant mechanism in the breakdown of the BBB, which contributes to the metastatic dissemination of leukemic cells in CNS leukemia.

## Materials and Methods

### Ethics statement

All animal work was done following an institutionally approved protocol in accordance with National Health Guides for the Care and Use of Laboratory Animals and the Law of Animal Experiments regulation of the University of Soochow (26/2008). All surgery was performed under chloral hydrate anesthesia, and all efforts were made to minimize suffering.

### Mice

Four-week-old male BALB/c nu/nu mice were purchased from the Shanghai Laboratory Animals Center of the Chinese Academy of Science and maintained under specific pathogen-free conditions. All mice were pretreated as follows: splenectomized on day 0, 2.5 mg cytoxan intraperitoneal injection for three days, surgery after seven days and total body sublethal irradiation of 4.0 Gy at rate of 2 Gy/min per dose on day 10. Those mice that received splenectomy, cytoxan intraperitoneal injection, and were pretreated with sublethal irradiation (SCI), were referred to as SCI-nu/nu mice.

### Cell culture, reagents and treatments

Leukemic cell line SHI-1 was originally derived from the bone marrow mononuclear cells of a patient with acutemonocytic leukemia in relapse, and maintained as an in vitro cell line [22]. SHI-1, NB4 and U937 cells were available and carried in our laboratory. Cells were maintained in Iscove's Modified Dulbecco's Medium (IMDM) (Gibco, USA) with a 10% fetal bovine serum (FBS) (Gibco, USA) at 37°C in a humidified atmosphere containing 5% CO_2_.

Primary human BMVECs were purchased from the American Sciencell Research Laboratory and cultured in a complete serum of endothelial cell medium (Sciencell, USA) in tissue culture flasks pre-coated with human fibronectin (3 mg/cm, Gibco, USA). Endothelial cells achieved 70∼80% confluence after 5 to 7 days. Following two passages digested with trypsin/EDTA solution, the cells were used to set experiments.

The broad-spectrum MMP inhibitor GM6001 (Calbiochem, Germany) was dissolved in dimethyl sulfoxide (DMSO) as a stock solution and further supplemented in PBS immediately before administration or in IMDM before incubation with leukemic cells. For inhibition studies, cells were incubated with 20 µg/ml (the concentration that highly inhibited the activities of MMP-2 and -9 in leukemic cells but had no influence on cell viability) GM6001. Cell viability was analyzed by trypan staining, which indicated cell survival rates of >95%.

### Transfection of SHI-1 cells with siRNA

MMP-2 and -9 genes of SHI-1 cells were knocked down by RNAi. A specific siRNA of MMP-2 was designed given the protocol suggested by Ries *et al* [23]. A specific siRNA of MMP-9 was designed by Genepharma (China). The sequences were as follows: sense, 5′-GAC CUG AGA ACC AAU CUC ATT-3′; antisense, 5′-UGA GAU UGG UUC UCA GGU CTT-3′. All oligonucleotides were synthesized by Genepharma. SiRNA transfection of SHI-1 was performed as previously described by us [24].

### Zymography

The activities of MMP-2 and -9 in the conditioned medium were determined by gelatin zymography as previously described [25]. Supernatants of different leukemia cell lines treated or untreated with GM6001, as well as the RNAi-treated cells for 24 h after transfection at the final density of 2.0×10^5^ cells/well suspended in serum-free medium (IMDM 100 µl) were seeded in 96-well plates. Supernatants were harvested after 24 h cultivation and 15 µl serum-free medium were collected and clarified by centrifugation to remove cells and debris. Samples were loaded under nonreducing conditions onto SDS-polyacrylamide gel polymerized with 1 mg/ml gelatin. Following electrophoresis, the gels were washed with 2.5% Triton X-100 to remove SDS and then incubated in a developing buffer [50 mM Tris–Cl pH 7.5, 10 mM CaCl2, 150 mM NaCl, 1 µM ZnCl2, 0.02%NaN3] overnight at 37°C. Gels were stained with 0.25% Coomassie Brilliant Blue R-250 and destained in the same solution without dye. Gelatinase activity was visualized as clear bands against the blue-stained gelatin background and analyzed in a computer system. Three individual experiments were conducted with independent protein samples.

### *In vitro* BBB model and evaluation of the barrier integrity

An *in vitro* BBB model composed of BMVECs was developed on a matrigel-based insert with 8.0 µm pore size in a 24-well plate. Growth-arrested BMVECs of 2×10^4^ were seeded onto the upper surface of matrigel and fibronectin-coated polycarbonate membranes in transwell culture chambers. After the cells were incubated for 5–8 d to allow cell attachment, we carried out our experiments. The permeability of the barrier and the formation of the monolayer and its tightness were judged by measuring dextran-FITC (100 µg/ml, 40 Kd, Molecular Probes) flux across the barrier by flow cytometry analysis. The matrigel-based insert without BMVECs was used as control. When the dextran-FITC became absent in the lower compartment, the model was used to set experiments.

### Invasion and cell migration assay

Twenty-four-well transwell inserts with 8 µm pores coated with matrigel (50 µL/well; BD Biosciences, Palo Alto, CA) were used to assess cell invasion. Different leukemia cell lines treated or untreated with GM6001, as well as the RNAi-treated cells for 24 h were suspended in 200 µl serum-free IMDM medium and seeded at a final density of 2.0×10^5^ cells/well in triplicate in the upper chamber. The lower compartment of the invasion chamber was filled with 800 µl IMDM (supplemented with 10% human serum) containing 100 ng/ml SDF-1a as a source of chemoattractants. The coated filter inserts with or without seeded BMVECs (2.0×10^4^ cells) were placed into the wells forming the upper compartment. The chambers were incubated for 24 h at 37°C in a 5% CO_2_ atmosphere. Cells that had migrated into the lower compartment were counted and the invasion rate was calculated by the ratio of the number of cells in the medium of lower compartment to the total number of leukemic cells loaded in the upper compartment. Morphologic changes of BMVECs on matrigel were observed under inverted phase contrast microscopy after washed with PBS. Photographs were captured using a camera (Canon A1000, Malaysia). Each invasion experiment was performed in triplicate.

### Murine CNS leukemia model and drug administration

For CNS leukemia animal model, a total of 1.2×10^7^ SHI-1 cells in 0.01 M PBS for a total volume of 200 µl were inoculated into 46 SCI-nu/nu mice *via* the dorsal tail vein after receiving sublethal irradiation. 46 SCI-nu/nu mice were randomized into 4 groups: 30 SCI-nu/nu mice were only inoculated with SHI-1 cells, 8 SCI-nu/nu mice were injected intraperitoneally with GM6001 (1.5 mg per mouse) daily until day 21, another 6 SCI-nu/nu mice were injected with PBS without SHI-1 cells and 2 mice were injected with the vehicle DMSO as control. Mice were monitored daily for survival and clinical appearance of nerve palsy and killed when becoming moribund. In order to identify the initial time of leukemic cells entering the CNS, 2 mice inoculated with SHI-1 cells and one control mouse were sacrificed weekly after inoculation.

### Reverse transcription-polymerase chain reaction (RT-PCR) analysis and quantitative real-time polymerase chain reaction (qRT-PCR)

Animals were anaesthetized with chloral hydrate and perfused with PBS prior to sacrifice in order to avoid RNA contamination from blood cells. Brains were immediately removed after perfusion and total RNA was extracted using Trizol reagent according to the manufacturer's instruction, and cDNA were synthesized by using MMLV reverse transcriptase (Gibco, USA). The oligonucleotide primer sequences were designed as the follows: human MMP-2, forward 5′-GCT ATG GAC CTT GGG AGA A-3′, reverse 5′-TGG AAG CGG AAT GGA AAC-3′, product 260 bp; human MMP-9, forward 5′-TCC CTG GAG ACC TGA GAA CC-3′, reverse 5′-CGG CAA GTC TTC CGA GTA GTT T-3′, product 287 bp; human GAPDH, forward 5′- AGA AGG CTG GGG CTC ATT TG -3′, reverse 5′- AGG GGC CAT CCA CAG TCT TC -3′,product 258 bp. MLL/AF6 fusion genes were detected by nested PCR performed as previously described [26]. The amplified products were analyzed by 2% agarose gel electrophoresis and photographed under UV light.

Levels of MMP-2 and MMP-9 mRNA were analyzed with the TaqMan Gene Expression Assays (Applied Biosystems). Glyceraldehyde-3-phosphatedehydrogenase (GAPDH) housekeeping gene was used as an endogenous control to correct potential variation in RNA loading or efficiency of the amplification procedure. Primers and probe specific for human MMP-2, MMP-9 and GAPDH used as reported previously [24], [27]. Real-time PCR was performed on a MJ Research OpticonTM2. The PCR thermal cycle conditions and calculation of the relative expression of mRNA species were performed as described previously [24]. All samples were measured in triplicate.

### Histology and immunofluorescence

Bone marrow smears of mouse femurs were stained with Wright's-Giemsa for observing leukemic blast cells. Brains were immediately removed after perfusion with 4% paraformaldehyde (PFA) in PBS, pH 7.4, fixed for 3 h in 4%PFA in PBS at 4°C, and incubated overnight in PBS with 20% sucrose at 4°C. Brains were cut into 6 or 20 µm thick sections with a cryostat. Sections of 6 µm thick were stained with hematoxylin and eosin (H&E). The remaining slides were used for immunofluorescence.

Immunofluorescence analysis determined the changes of TJ proteins in brain sections and BMVECs on Matrigel. Cell monolayers cultured on inserts were washed in PBS and fixed with 4% PFA. After blocking with 1% or 5% BSA, cells or brain sections were stained with primary monoclonal antibodies against ZO-1, occludin and claudin-5, conjugated to FITC or Alexa 488 (Invitrogen, USA) overnight at 4°C. The primary antibodies were used in a 1∶50 dilution. For double labels, anti-fibrinogen (1∶200, Abcam, USA) goat monoclonal antibody or anti-CD31 (1∶50, Abcam, USA) rat monoclonal antibody were incubated with these primary antibodies respectively. Incubation with the secondary antibody Cy3-labelled anti-goat or anti-rat IgG (1∶750) lasted for 1 h at 37°C. Nuclei were stained with DAPI (Molecular Probes, Invitrogen) for 3 min. Staining was analyzed with a laser-scanning confocal microscope (Nikon A1, Japan).

### *In situ* gelatin zymography

The frozen, nonfixed sections from brain tissues were incubated in a humidity chamber for 3 h at 37°C in a reaction buffer (0.05 M Tris–HCl, 0.15 M NaCl, 5 mM CaCl, 0.2 mM NaN3) containing 20 mg/ml of fluorescein isothiocyanate (FITC)-labeled DQ-gelatin (EnzCheck Collagenase Kit, Invitrogen, USA). As a negative control, 50 µM of 1, 10-phenanthroline, a metalloproteinase inhibitor, was added to the reaction buffer before applying the FITC-labeled DQ gelatin to frozen sections. After incubation, fluorescence was detected by laser scanning fluorescence confocal microscopy. Quenched FITC molecules contained within the gelatin emit fluorescence when the gelatin is enzymatically cleaved by MMP-2/-9.

### Immunoblotting

Equal amounts of protein (80 µg) were loaded on 6 or 10% polyacrylamide gels and SDS-PAGE was performed. After separation by electrophorese, proteins were electroblotted on polyvinyl difluoride membranes. The membranes were incubated with primary monoclonal mouse antibodies against against ZO-1 (2 µg/ml; Inritrogen), Claudin-5 (3 µg/ml; Inritrogen), occludin (2.5 µg/ml; Inritrogen) at 4°C overnight. After the blots were incubated with secondary antibodies, bands were visualized by using the ECL chemiluminescence detection system. The experiment was repeated at least twice with independent protein samples.

### Data analysis

Statistical analysis was carried out using ANOVA followed by Tukey's post hoc tests (GraphPad 4.0 Software, San Diego, CA). Significance was assumed for *p* value less than 0.05.

## Results

### MMP-2 and -9 are expressed by different leukemic cell lines and correlates with their invasive capacity

**Figure 1:**
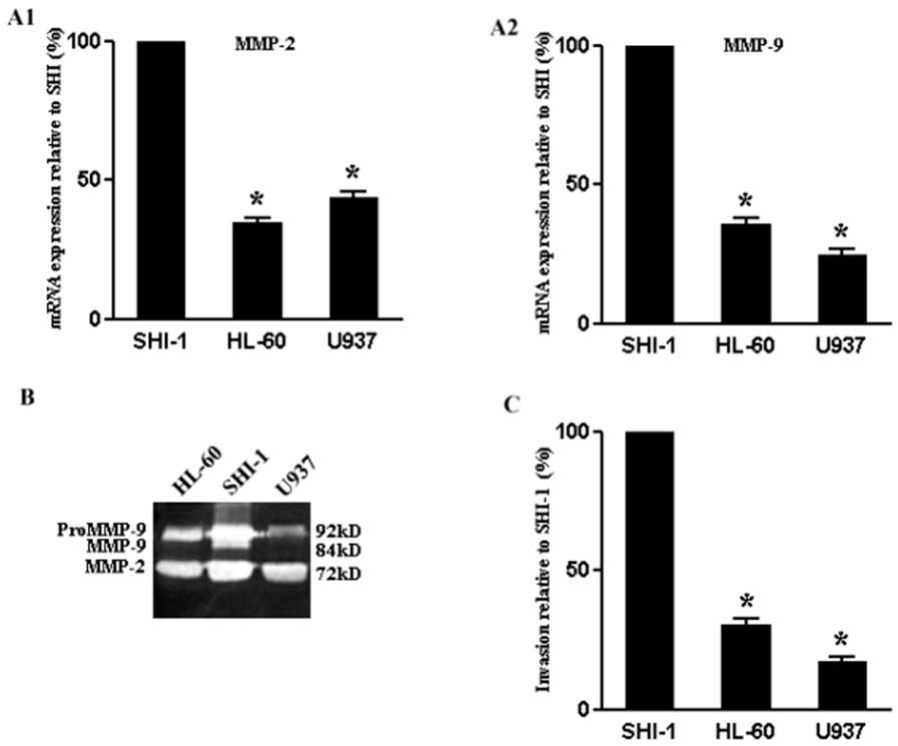
Expression of MMP-2 and -9, gelatinase secretion and invasion rate in different leukemic cells. (A) qRT-PCR analysis of MMP-2 (A1) and MMP-9 (A2) gene transcription in SHI-1, HL-60 and U937 leukemic cells. (B) Gelatinase secretion from culture supernatants of three leukemic cells. (C) Cell invasion rates of SHI-1, HL-60 and U937 leukemic cells. Cell invasion rates were determined by the ratios relative to SHI-1 cells. Data represent the mean ± SD of triplicate measurement representative for three independent experiments relative to SHI-1 cells (set as 100%). Compare with SHI-1 cells *P<.001.

The qRT-PCR determination of mRNA expressions in SHI-1, HL-60 and U937 cells revealed that the transcriptional level of MMP-2 and -9 in SHI-1 cells were all markedly higher than that in other leukemic cell lines (*P*<.001) (Fig. 1A). Furthermore, the zymographic analysis of MMP-2 and -9 activities of the cell culture supernatants were consistent with their mRNA levels. Moreover, SHI-1 cells released a small portion of activated MMP-9 into culture supernatants (Figure 1B). Cell migration assay was used to measure the invasive capacity of leukemic cells. The invasion rate of SHI-1 was significantly higher than that of HL-60 and U937 cells (*P*<.001) (Figure 1C).

### Increased permeability of the BBB by leukemic cells and down-regulation of endothelial TJ proteins via MMP-2 and -9 *in vitro*

**Figure 2:**
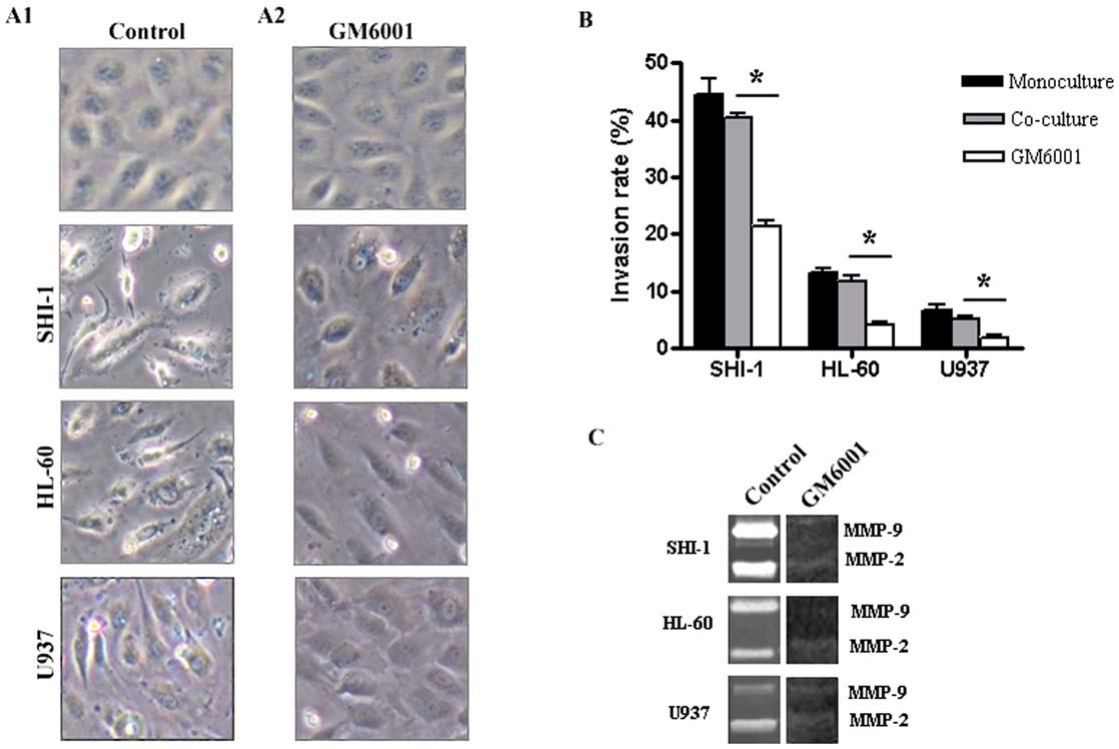
Increased *in vitro* BBB permeability due to MMP-2 and -9 secreted by SHI-1, HL-60 and U937 cells. (A) Morphological changes of BMVECs affected by SHI-1, HL-60 and U937 cells treated with or without GM6001. (B) Rates of leukemic cell invasion of co-culture with BMEVCs compared with those of monoculture (without BMEVCs) (*P*>.05) and those treated with GM6001 (*P<.001). Results are shown as the mean ± SD of three independent experiments. (C) Zymography gelatinase secretion in SHI-1, HL-60 and U937 cells treated with or without GM6001. Original magnification: (A)×600.

We hypothesized that MMP-2 and -9 secreted by leukemic cells induced the permeability of the BBB by disrupting the TJ proteins in CNS leukemia. To test this hypothesis, we first seeded SHI-1, HL-60 and U937 cells into the upper compartment of the *in vitro* BBB model, constituted by primary human BMVECs to form a co-culture system. The results showed that before co-culture with SHI-1, HL-60 and U937 cells, BMVECs became confluent with neighbouring cells tightly packed against each other leaving no gaps. However, after incubation for 24 h with leukemic cells, the tightness of the BMVECs was disrupted, giving rise to a loss of cell-cell contact and the formation of intercellular gaps (Fig. 2A1). The number of leukemic cells across the barrier was calculated as the rate of invasive in order to reflect the degree of BBB breakdown. The rates of invasion of leukemic cells in this co-culture system were inconsistent with their secreted MMP-2 and -9 activities and their ability to invade. Where SHI-1 showed a higher invasion capacity than either HL-60 or U937 cells (Fig. 2B). Interestingly, the degree of morphological changes in BMVECs depicted in Figure 2A1 matched well with the rate of invasive of different leukemic cells. SHI-1 cells caused the strongest disruptive effects on BMVECs and the BBB.

To further determine whether MMP-2 and MMP-9 secreted by leukemic cells played roles in increasing the permeability of BMVECs and the BBB, SHI-1, HL-60 and U937 cells were treated with the MMP inhibitor GM6001, which had been previously shown to inhibit MMP-2 and -9 [27], before co-culture with BMVECs in the *in vitro* BBB model. We found that the intercellular gap of BMVECs, led by leukemic cells, became tight (narrow) or even partially disappeared (Fig. 2A2). Consistently and significantly, the number of leukemic cells across the BBB decreased, reflecting a decided reversal in the impairment of the BBB. Gelatin zymography of supernatants from GM6001-treated leukemic cells demonstrated a distinct loss of MMP-2 and -9 enzyme activities, compared to the untreated supernatants, thereby confirming the ability of GM6001 to block MMP-2 and -9 activities (Fig. 2C). These results show that MMP-2 and -9 secreted by leukemic cells were closely related to the BBB breakdown by disrupting BMVECs.

**Figure 3:**
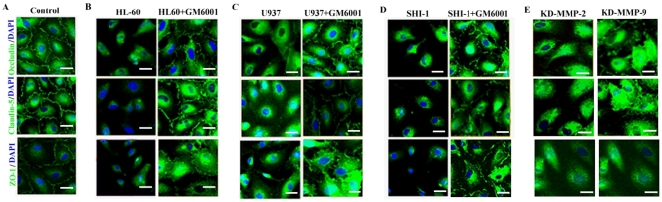
Confocal imaging showed protein localization of ZO-1, claudin-5 and occludin in human BMEVCs. (A) Confocal imaging of ZO-1, claudin-5 and occludin of human BMEVCs; (B, C, D) BMEVCs co-cultured with SHI-1, HL-60 and U937 cells with or without treatment by GM6001. (E) BMEVCs of co-cultured with SHI-1 cells after knock-down of the MMP-2/MMP-9 gene. Data in all panels are representative of at least 3 separate experiments on 3 distinct cultures. (Scale bars: 10 µm).

To define whether the breakdown of BBB induced by MMP-2 and -9 was led by changes in TJ in addition to the basement membrane, BMVECs were immunostained for the TJ proteins ZO-1, claudin-5 and occludin. Confocal imaging demonstrated that in human BMVECs cultures, these proteins remained were localized on the plasma membrane in areas of cell-cell contact (Fig. 3A). After incubation with leukemic cells, the fluorescent immunoreactivity for ZO-1, claudin-5 and occludin appeared patchy or even faded away in those areas accompanied by the same degree of BBB breakdown (Fig. 3B–D). On the contrary, the expression of three proteins, all up-regulated in company with the decreased BBB permeability when MMP-2 and -9 were secreted by leukemic cells was inhibited by GM6001 (Fig. 3B–D).

Collectively, these results suggested that leukemic cells disrupted the BBB by down-regulating the TJ proteins ZO-1, claudin-5 and occludin in BMVECs. MMP-2 and -9 secreted by leukemic cells play important roles in this process.

### SHI-1 cell's invasion impaired by knock-down of MMP-2 or -9 genes and decreased disruption of ZO-1, claudin-5 and occludin with alleviative permeability of *in vitro* BBB

**Figure 4:**
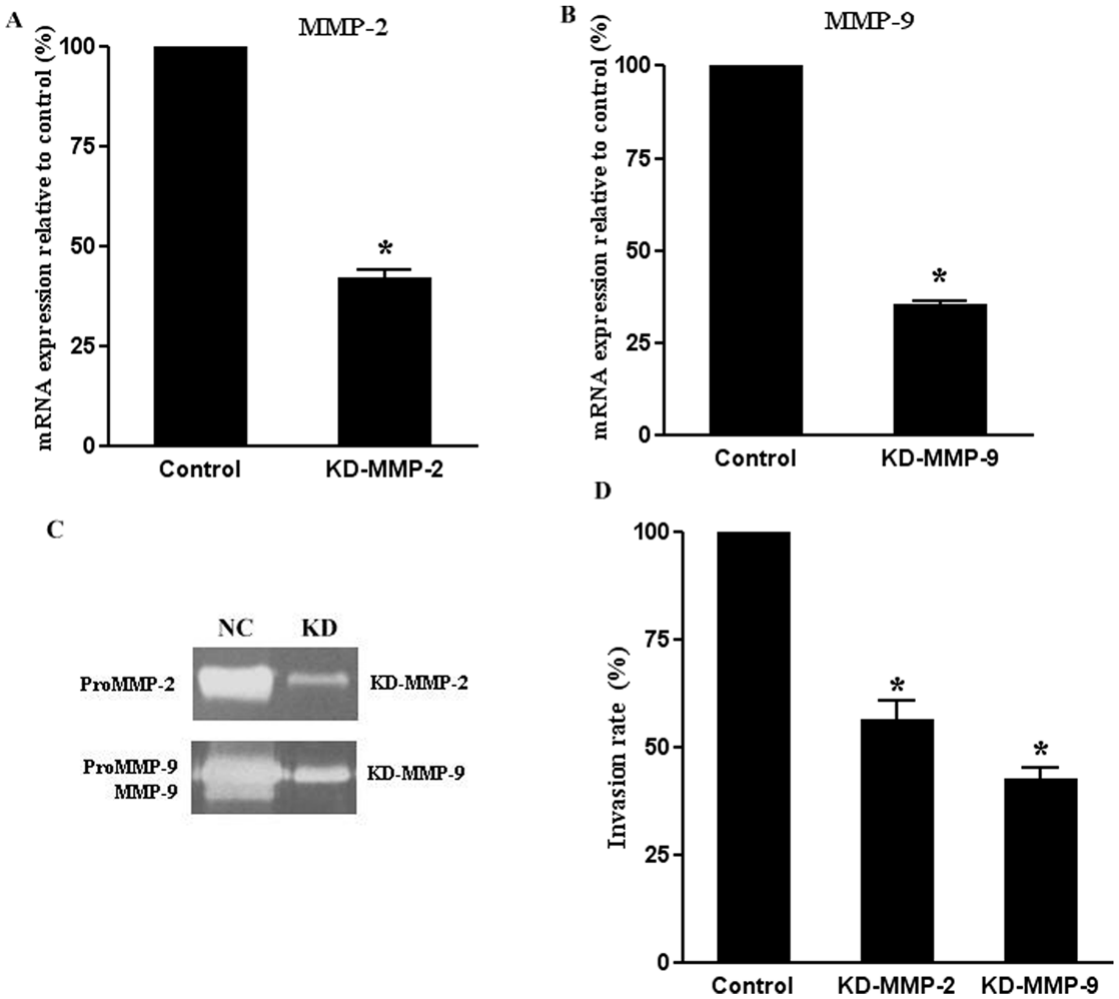
Effects of MMP-2/MMP-9 knock-down on activity of gelatinase and rates of cell invasion by SHI-1 in the *in vitro* BBB model. (A) Transcription of specific mRNAs quantified by qRT-PCR 48 h after siRNA transfection. SHI-1 cells transfected with siRNAs targeting the expression of MMP-2 (KD-MMP-2) or (B) MMP-9 (KD- MMP-9). Control SHI-1 cells transfected with non-target-directed siRNA (set as 100%). (C) Gelatinase secretion from SHI-1 cells transfected with siRNAs. (D) Rates of cell invasion of SHI-1 cells 48 h after transfection with siRNA in in vitro model of BBB. Data represent the mean ± SD of triplicate measurement representative for three independent experiments relative to SHI-1 cells (set as 100%). *P<.001.

To elucidate the specific contribution of MMP-2 and -9 in the role of the BBB opening, we silenced the genes expression of MMP-2 and -9 in SHI-1 cells by means of RNAi. The down-regulation of the mRNA expression was still effective with levels between 60% and 65% when determined 24 h after treatment with the respective siRNAs (Figure 4A). These results were confirmed by zymographic analysis of cultured supernatants and demonstrated successful blockage of MMP-2 and -9 productions (Figure 4B). Down-regulation of MMP-2 and -9 also impaired the invasive capacity of SHI-1 cells (data not shown). Furthermore, to examine the BBB disruption related to MMP-2 and -9 secreted by SHI-1 cells, RNAi-treated SHI-1 cells were cultured with BMVECs in the *in vitro* model of BBB. As shown in Fig. 4C, the rate of invasion of SHI-1 cells reduced when MMP-2 or -9 genes were knocked-down, which showed the relative weaker disruption of the BBB (*P*<.001). Consistently, immunofluorescence for ZO-1, claudin-5 and occludin showed the knock-down of MMP-2 or -9 gene partially relieved the degradation of TJ proteins (Fig. 3E). The morphological changes of BMEVCs also demonstrated this phenomenon (data not shown) Thus, these findings demonstrate that down-regulation of gene expression of MMP-2 or -9 in SHI-1 cells could partially reverse the disruption of ZO-1, claudin-5 and occludin and so decrease the permeability of the BBB. These results further confirm the roles of MMP-2 and -9 in disrupting the BBB by degrading TJ proteins.

### Fatal CNS leukemia in SCI-nu/nu mice caused by the human acute monocytic leukemia cell line SHI-1

**Figure 5:**
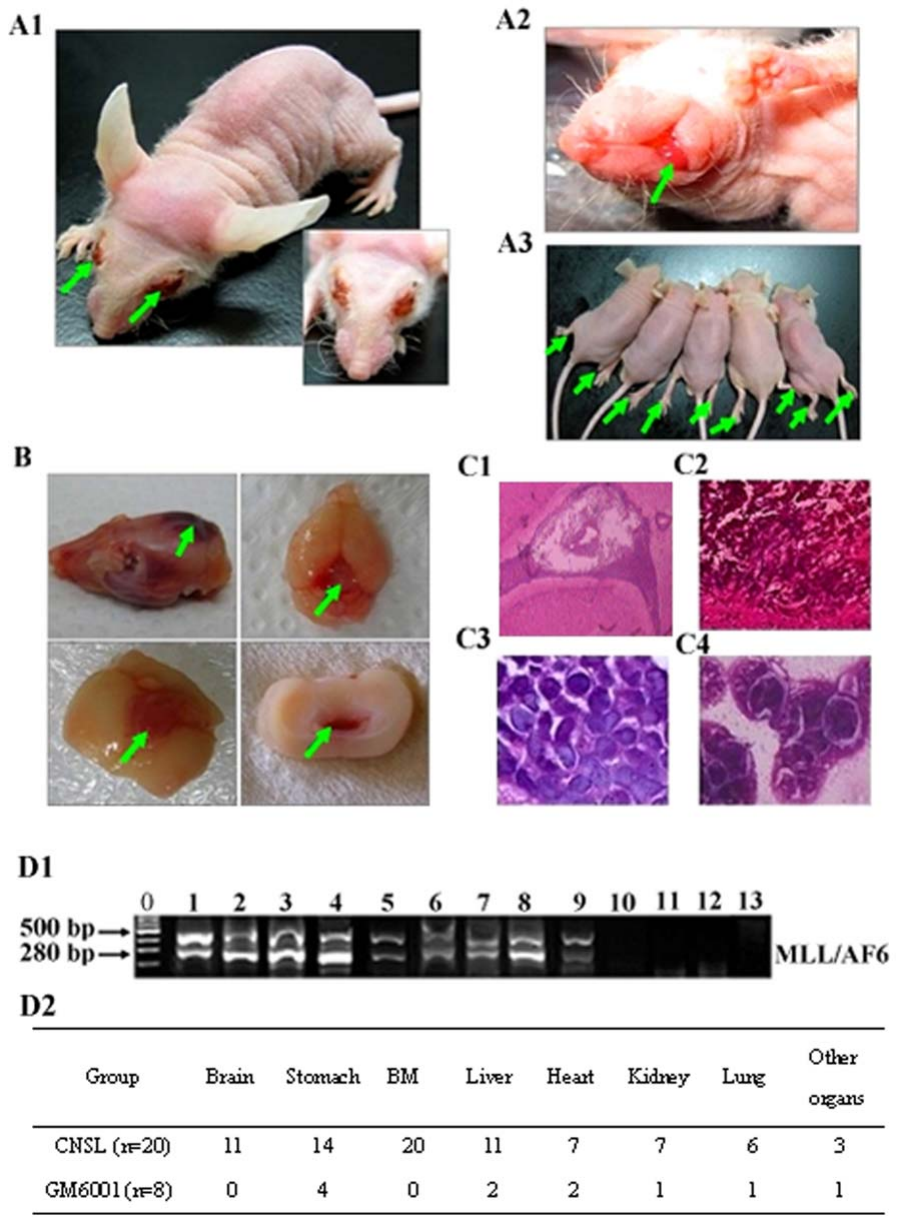
The SCI-nu/nu mice model of CNS leukemia. (A) Clinical signs of CNS leukemia in mice. (A1, A2, arrow) The mouse developed paralysis in its left forelimb and cranial nerve with light loss. (A3, arrow) The mice developed paralysis in both fore limbs and hind limbs. (B, arrow) Neoplasms of different parts with or without intracranial hemorrhage in removed brains. (C) Infiltrations of SHI-1 examined with histopathology assay. (C1, C2, C3) Brain parenchyma pathobiology stained with H&E. (C4) Examination of bone marrow smears stained with Wright's-Giemsa. (D) Organs with infiltrations of SHI-1 examined with RT-PCR analysis of human MLL/AF6 fusion gene transcription in mice with or without GM6001 treatment. (D1) RT-PCR analysis of MLL/AF6 fusion gene of brains and bone marrow from SCI nu/nu-mice. Lanes 1–5: Human MLL/AF6 fusion genegene amplified in SHI-1 cell and in brain, bone marrow, stomach and liver of mice with CNS leukemia. lane 6–9: Human MLL/AF6 fusion gene amplified in stomach, liver, brain and bone marrow of mice with GM6001 treatment. lane 10: negative control. (D2) The number of organs with infiltrations of SHI-1 examined with RT-PCR analysis of human MLL/AF6 fusion gene transcription in mice with or without GM6001 treatment. Original magnification: (C1), ×40; (C2), ×200; (C3), ×1000; (C4), ×1000.

In order to investigate whether the BBB breakdown in CNS leukemia was associated with changes in endothelial TJ proteins *in vivo*, we next established a mouse model of CNS leukemia with the highly invasive human acute monocytic leukemia cell line SHI-1. Of the 20 surviving mice (other 10 mice were sacrificed weekly) challenged with SHI-1 cells, 11 mice developed symptomatic CNS leukemia. The prominent clinical sign of CNS leukemia was muscle weakness in the legs with a slight lowering of the lower back.. Subsequently, mice developed a rapidly progressive paralysis of the cranial nerve with or without sight loss and they were no longer able to obtain food or water at (32.55±0.65) d (mean ±SD) after being challenged by SHI-1 (Fig. 5A). They were killed when mice were moribund. Intracerebral mass or chloroma with or without intracranial hemorrhage was found in the removed brains (Fig. 5B). Histopathology examination showed that the CNS was extensively involved in these mice, with leukemic cell infiltrating the brain parenchyma (Fig. 5C1–C3) and subarachnoid spaces or accumulated on the surface of the cerebrum (data not shown). Bone marrow smear demonstrated a lot of leukemia blasts, infiltrating the bone marrow (Fig. 5C4). Histopathology also demonstrated the infiltration by SHI-1 cells in livers, lungs, kidneys, testes, hearts and other organs (data not shown).

SHI-1 cells have t (6; 11) (q27; q23) and the rearrangement of the MLL gene, expressing the MLL/AF6 fused gene product [22]. MLL/AF6 was initially amplified in the brain at day 21 after challenged SHI-1 in some mice. The transcription of MLL/AF6 fusion gene also could be detected in the brains and bone marrows of killed mice with paralysis (Fig. 5D1). In control mice and mice without CNS leukemia, we did not examine for MLL/AF6 fusion gene in brains. All of our data demonstrate that the animal model of CNS leukemia with brain parenchyma involvement was successfully established with the human acute monocytic leukemic cell line SHI-1.

### GM6001 protected mice against CNS leukemia

**Figure 6:**
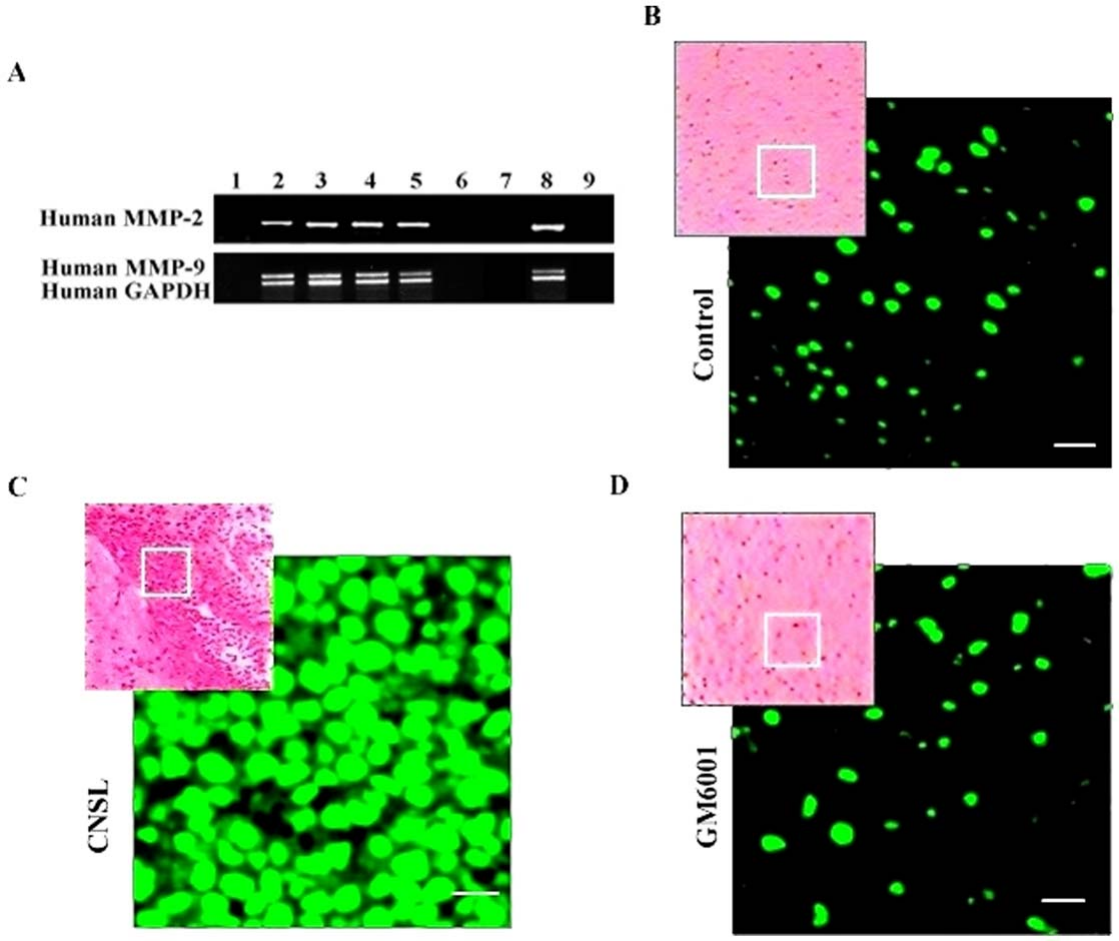
Activities of MMP-2 and -9 secreted by SHI-1 with or without GM6001 treatment in CNS leukemia mice. (A) RT-PCR analysis of human MMP-2 and -9 gene transcription in normal brain tissues, brain tissues infiltrated with SHI-1 cells with or without GM6001 treatment. (B, C and D) *In situ* zymographic analysis of MMP-2 and -9 activities mouse tissues. (B) Normal brain tissues; (C) Brain tissues infiltrated with SHI-1 cells; (D) Brain tissues after treatment with GM6001. Results shown are from individual animals and are representative of findings from 3 experiments with 6 animals per condition per experiment. (Scale bars: 10 µm).

Consistent with MLL/AF6, the human MMP-2 and -9 transcriptions were initially detected at day 21 after being challenged by SHI-1 and amplified in all killed mice with CNS leukemia (Fig. 6A). In order to examine the roles of MMP-2 and -9, GM6001 was used to inject intraperitoneally into mice. We found that no mice developed CNS leukemia until they were killed when moribund at (41.50±1.62) d (mean ±SD) after being challenged by SHI-1. The number of organs infiltrated by SHI-1 cells and the degree of infiltration also decreased (Fig. 5D). MMP-2 and -9 transcripts were not detected in the brains of mice treated with GM6001. (Fig. 6A). Furthermore, we used *in situ* zymography to identify the regions with gelatinolytic activity. Bright fluorescent (green) spots were apparent in the areas of infiltration with leukemic cells and in the periphery of mouse brains with CNS leukemia (Fig. 6C). While after treatment with GM6001, gelatinolytic activities had not changed in all mice compared with normal control mice, fewer fluorescent spots were seen in these brain sections (Fig. 6B, D). These data demonstrate that MMP-2 and -9 secreted by SHI-1 cells play important roles in leukemic cell infiltration to CNS and other extramedullary infiltration.

### BBB breakdown with the disruption of endothelial TJ proteins in CNS leukemia and GM6001 reversed these changes

**Figure 7:**
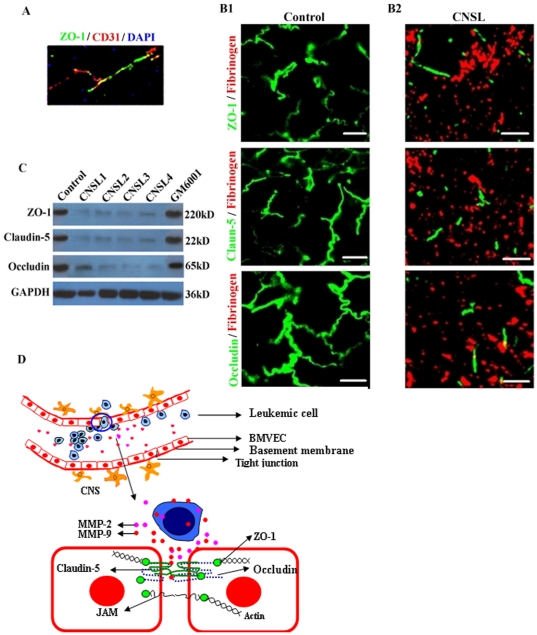
Increased BBB disruption, with a decrease in endothelial ZO-1, claudin-5 and occludin, avoided by GM6001. (A) Confocal imaging of cerebral cortex from normal mice immunostained for ZO-1 localize at vessels, confirming the use of CD31. (B1, B2) Confocal imaging of TJ proteins ZO-1, claudin-5, occludin and fibrinogen in brain parenchyma in control and CNS leukemia mice. (C) Immunoblots of protein extracts from brains of control mice, 4 CNS leukemia mice with brain parenchyma and CNS leukemia mice treated with GM6001. In all figures, brain tissues of normal mice used as control. (D) Model of MMP-2 and -9 secreted by leukemic cells disrupting several key TJ proteins of BMVECs in BBB breakdown. Results shown are from individual animals and are representative of findings from 3 experiments with 4 animals per condition per experiment. (Scale bars: 10 µm).

To investigate whether BBB breakdown in CNS leukemia is associated with changes in endothelial TJ proteins, brain tissue sections were immunostained for ZO-1, claudin-5 and occludin, plus fibrinogen as a marker of the BBB disruption. Fibrinogen is a serum protein which is absent in the CNS under normal physiological conditions. Confocal imaging of the brain cortex from normal control mice showed that ZO-1, claudin-5 and occludin localized to linear profiles, were confirmed as vessels using the CD31 antigen (Fig. 7A, B1). In brains of mice with leukemic cell infiltration, confocal imaging showed widespread BBB breakdown as assessed by parenchymal immunoreactivity for fibrinogen (Fig. 7B2). Importantly, ZO-1, claudin-5 and occludin immunoreactivity also appeared reduced in these areas (Fig. 7B2). Moreover, immunoblotting for ZO-1, claudin-5 and occludin showed that the protein levels were down-regulated in brains with CNS infiltration than in normal mice brains (Fig. 7C). However, these three TJ proteins did not change in the brain of mice with GM6001 treatment compared with normal control mice (data not shown). In accordance with the results of immunoreactivity, the protein levels did not change after MMP-2 and -9 were inhibited by GM6001 (Fig. 7C). Collectively, these results demonstrate that the loss of endothelial TJ proteins ZO-1, claudin-5 and occludin leaded by MMP-2 and -9 is a central event in the opening of the BBB in CNS leukemia.

## Discussion

In this study, we have investigated the mechanism underlying the BBB breakdown which makes leukemic cell enter the CNS during the process of leukemic cell extramedullary infiltration. We conclude that MMP-2 and -9 secreted by leukemic cells have been implicated in the response. The targets of the MMP-2 and -9 are the endothelial TJ proteins in addition to the well-known ECM. MMP-2 and -9 distinguishably disrupted the TJ proteins ZO-1, claudin-5 and occludin as the BBB breakdown. We also have shown that these alterations can be inhibited by the broadband MMP inhibitor GM6001 and the knock-down of MMP-2/MMP-9 genes. Furthermore, GM6001 could effectively protect the mice against CNS leukemia. These findings may provide new insights into the mechanism of CNS involvement in leukemia.

The invasion process of cancer cells involves the secretion of substances to degrade the components of the extracellular matrix, as well as the proteins involved in the control of motility and migration [28]. It is well-known that MMP-2 and -9 play critical roles in solid tumor invasion. For leukemia, the blasts circulating in the bloodstream, must have different invasive characteristics. Our previous study and other evidence implicated abnormal expression of MMP-2 and -9 in AML and their correlation with the extramedullary infiltration [15], [16], [27]. Reikvam H *et al*. reported that MMP-9 release was higher in AML cells with monocytic differentiation corresponding to the FAB-subtype M4/M5 AML [29]. Paupert *et al*. also found that a surface-bound MMP-9 form may contribute to the invasive capacities of monocytic leukemia (M4 and M5) cells *in vitro* and their extramedullary infiltration including CNS leukemia *in vivo* [30]. The continuous complexes of TJs combined with reduced vesicular transport make the BBB are much difficult to penetrate, and infiltration through these barriers selects for leukemic cells that express the necessary extravasation functions. Although reports have shown that MMP-2 and -9 are closely associated with the BBB disruption in some CNS related diseases [20], [31], little is known about the pathological role of MMP-2 and -9 secreted by leukemic cells in BBB breakdown in CNS leukemia.

In order to investigate the relationship between leukemic cell-derived MMP-2 and -9 and the BBB in CNS leukemia, we first studied the effect of MMP-2 and -9 secreted by different leukemic cells on cultured BMVECs in the *in vitro* model of the BBB. Before transmembrane of leukemic cells, we seeded BMVECs onto the upper surface of matrigel-coated polycarbonate membranes in transwell culture chambers to simulate the barrier of the BBB. Cultured BMVECs retain many of the features of the BBB and have been used as *in vitro* models to study mechanisms that regulate the permeability of the BBB [32], [33]. It is well-known that BBB is maintained primarily by TJs between adjacent microvascular endothelial cell images as a network of strands formed by intramembranous particles and occlude intercells more effectively [10], [11], [34]. The transmembrane proteins occludin and claudin-5 form the primary seal of the TJs. They bind to the ZO-1 and other proteins that couple the TJs to the actin cytoskeleton of endothelial cells [10]. Studies have demonstrated a correlation between the reduction of TJ and tumor differentiation and placed TJ in the frontline as the structure that cancer cells must overcome in order to metastasize [28], [35], [36]. We also found that each of the different leukemic cell lines HL-60, SHI-1 and U937 all could disrupt the TJ proteins ZO-1, claudin-5 and occludin, accompanying the formation of intercellular gaps in co-cultured BMVECs and the breakdown of BBB, but the levels of disruption differed greatly. SHI-1 cells displayed a stronger capacity of disrupting TJ proteins than the other two leukemic cell lines, consistent with its higher rate of invasion which is closely related to the constitutive strong expression of MMP-2 and -9. GM6001 is a broad-spectrum MMP inhibitor to prevent the activities of MMP-2, -3, -8 and -9 and is routinely used to understand the pathogenic roles of MMP-2 and -9 [23], [37]. We have shown that GM6001 blockaded the activities of MMP-2 and -9 and significantly decreased the disruption of the leukemic cells to the TJ proteins. Moreover, the knock-down of MMP-2 or MMP-9 genes by iRNA in SHI-1 cells also reduce the disruption of those proteins and the permeability of BBB. These findings suggest that MMP-2 and -9 secreted by leukemic cells can degrade components of TJ in addition to basement membranes, which help the leukemic cells to infiltrate the brain.

Our previous study has created an efficient and reproducible experimental model of AML CNS leukemia and multiorgan infiltration by using highly invasive SHI-1 cells in nu/nu mice [26]. However, a mouse model of CNS leukemia can not be successfully established with HL-60 and U937 cells. In this study we increased the number of inoculated SHI-1 cells. More than half of the mice developed CNS leukemia and 4 of them had SHI-1 cell infiltration in the brain parenchyma. In the section of infiltrarion with SHI-1 cells, we found a widespread BBB breakdown with a loss of the endothelial TJ proteins ZO-1, claudin-5 and occludin. The accompanying up-regulation of human MMP-2 and -9 showed that MMP-2 and -9 secreted by SHI-1 cells played an important role in this event. Surprisingly, GM6001 protected mice against CNS leukemia and as well, other organs were protected and no mice developed CNS leukemia. These results further defined that MMP-2 and -9 secreted by leukemic cells increased the permeability of the BBB by disrupting the TJ proteins ZO-1, claudin-5 and occludin in CNS leukemia.

We also found that both MMP inhibitor GM6001 and the RNAi technology to inhibit the expression of MMP-2 and -9 in leukemic cells could not entirely reverse the disruption of the TJ proteins and the permeability of the BBB *in vitro*. In addition to MMP-2 and -9, other MMP family members such as MMP-1, MMP-3, MMP-11 and MMP-14 have all been shown to be secreted by tumor cells in tumor microenvironments, serving to mediate the breakthrough of tissue barriers comprising cell–cell adherent junctions, basement membranes, and interstitial tissue stroma [38]–[40]. Furthermore, the invasive behavior of tumor cells depends not only upon the secretion of a variety of degradative enzymes but also on the alterations in the expression of adhesion molecules (selectin–selectin ligand axis), the responses to cytokines and chemokines (CXCR4, CCR7, et al.) and the gene products regulating motility. Multiple mechanisms appear to contribute to the disease.

The integral transmembrane proteins such as claudin-5 and occludin are the essential adhesion proteins responsible for correct assembly of the TJ structure of the BBB and controlling TJ functions via homotypic and heterotypic interactions [10], [28]. There is an ongoing interest in the question of how the TJ proteins are modified by MMP-2 and -9 as it occurs in CNS leukemia. ZO-1, claudin-5 and occludin cleavage by MMPs is a possible mechanism underlying BBB impairment and has been detected *in vivo* models of focal cerebral ischemia [18], [41], [42]. Both occludin and claudin-5 consist of four transmembrane domains, intracellular N- and C-termini, and two extracellular domains that might interact with cell membranes of vicinal cells thus sealing the intercellular clefts [43], [44]. The first extracellular loop displayed type IV collagenase sensitive motives, implying that claudin-5 and occludin cleavage might be caused by MMP-2 or -9 [45], [46]. In addition to the most important functions as a barrier to cell migration, TJs have been linked to various signaling mechanisms [44], hence possible mechanisms will be further investigated.

In conclusion, our results established a correlation between MMP-2 and -9 secreted by leukemic cells and the disruption of the TJ proteins ZO-1, claudin-5 and occludin, which increases paracellular permeability of leukemic cell across the BBB in CNS leukemia. This information provides an important mechanism of leukemic cell infiltration of the CNS, which may provide additional insight into its potential target for therapeutic intervention. Future studies will have to clarify the precise mechanism or signal transduction pathway for the permeability-increasing effect of leukemic cell-derived MMP-2 and -9.
