# Correction: Matrix Metalloproteinase-2 and -9 Secreted by Leukemic Cells Increase the Permeability of Blood-Brain Barrier by Disrupting Tight Junction Proteins

**DOI:** 10.1371/annotation/716c0fb2-dbdd-4da5-ad8a-d2b1cdac4ec6

**Published:** 2011-08-23

**Authors:** Saran Feng, Jiannong Cen, Yihong Huang, Hongjie Shen, Li Yao, Yuanyuan Wang, Zixing Chen

The authors would like to add the following funding sources to their funding information: "This study is also supported by National Key Scientific Project of China (973 Program #2011CB933500) and the Priority Academic Program Development of Jiangsu Higher Education Institutions，PAPD." 

